# Exploring the Role of CBX3 as a Potential Therapeutic Target in Lung Cancer

**DOI:** 10.3390/cancers16173026

**Published:** 2024-08-30

**Authors:** Muhammad Aamir Wahab, Nunzio Del Gaudio, Biagio Gargiulo, Vincenzo Quagliariello, Nicola Maurea, Angela Nebbioso, Lucia Altucci, Mariarosaria Conte

**Affiliations:** 1 Department of Precision Medicine, University of Campania “Luigi Vanvitelli”, 80138 Naples, Italy; muhammadaamir.wahab@unicampania.it (M.A.W.); nunzio.delgaudio@unicampania.it (N.D.G.); biagio.gargiulo@unicampania.it (B.G.); angela.nebbioso@unicampania.it (A.N.); 2 Division of Cardiology, Istituto Nazionale Tumori-IRCCS-Fondazione G. Pascale, 80131 Naples, Italy; quagliariello.enzo@gmail.com (V.Q.); n.maurea@istitutotumori.na.it (N.M.); 3 Program of Medical Epigenetics, Vanvitelli Hospital, 80138 Naples, Italy; 4 Institute of Endocrinology and Oncology “Gaetano Salvatore” (IEOS), 80131 Naples, Italy; 5 Biogem Institute of Molecular and Genetic Biology, 83031 Ariano Irpino, Italy

**Keywords:** CBX3/HP1γ, lung cancer, epigenetics, chromatin and signaling pathways

## Abstract

**Simple Summary:**

Lung cancer is a major cause of cancer-related deaths in developed nations. Factors such as unhealthy lifestyle choices, particularly smoking, contribute to the development of this disease. Epigenetic abnormalities greatly affect gene expression and disrupt important cellular signaling pathways that are responsible for the proper growth, regulation, and functioning of cells. In the case of lung cancer, specifically non-small cell lung cancer (NSCLC), CBX3 acts as an epigenetic oncoprotein, promoting the growth and progression of tumors. Numerous studies have shown that CBX3 is overexpressed in NSCLC and is associated with a poor prognosis. It interacts with key oncogenic pathways leading to increased proliferation, reduced apoptosis, and enhanced resistance to therapy. Further research on the mechanisms and functions of CBX3 holds the potential to reveal new insights into the development of the disease and uncover novel therapeutic opportunities.

**Abstract:**

Epigenetic changes regulate gene expression through histone modifications, chromatin remodeling, and protein translation of these modifications. The PRC1 and PRC2 complexes shape gene repression via histone modifications. Specifically, the CBX protein family aids PRC1 recruitment to chromatin, impacting the progressive multistep process driving chromatin silencing. Among family members, CBX3 is a complex protein involved in aberrant epigenetic mechanisms that drive lung cancer progression. CBX3 promotes lung tumorigenesis by interacting with key pathways such as PI3K/AKT, Ras/KRAS, Wnt/β-catenin, MAPK, Notch, and p53, leading to increased proliferation, inhibition of apoptosis, and enhanced resistance to therapy. Given our current lack of knowledge, additional research is required to uncover the intricate mechanisms underlying CBX3 activity, as well as its involvement in molecular pathways and its potential biomarker evaluation. Specifically, the dissimilar roles of CBX3 could be reexamined to gain a greater insight into lung cancer pathogenesis. This review aims to provide a clear overview of the context-related molecular profile of CBX3, which could be useful for addressing clinical challenges and developing novel targeted therapies based on personalized medicine.

## 1. Introduction

Modifications associated with chromatin structure and function, known as epigenetic changes, are responsible for the activation and repression of genes, thereby impacting the synthesis and production of specific proteins within cells and with variable expression patterns [1,2,3]. Gene transcription can be reversibly modulated via chromatin remodeling, which regulates the accessibility of promoter and enhancer regions to regulatory proteins. The epigenetic regulatory complex, known as polycomb repressive complex (PRC) 1, is a chromatin-modifying complex that is responsible for keeping the chromatin in a repressed state by mono-ubiquitinating histone H2A, thus restricting target gene transcription [4,5]. PRC1 is completed via its association with the chromobox (CBX) family members, which are responsible for its recruitment to chromatin, whereas the RING1a/b subunit represents the catalytic subunits of the complex [6]. Similarly, PRC2 is composed of multiple subtypes of protein that collaborate with PRC1 to participate in the epigenetic regulation of gene expression. PRC2 has become a highly promising target for therapeutic intervention. The CBX family can be classified into two distinct groups. The first group, the polycomb group (PcG), consists of CBX2, CBX4, CBX6, CBX7, and CBX8, all of which contain a C-terminal polycomb repressor box and a conserved N-terminal chromodomain. The second group, the Heterochromatin Protein 1 (HP1) group, comprises CBX1, CBX3, and CBX5, characterized by an N-terminal chromodomain and a chromoshadow domain associated with HP1. CBX family proteins facilitate the recruitment of PRC1 to chromatin, thus playing a crucial role in the initiation, growth, and development of tumors by suppressing the differentiation of cancer stem cells and promoting their self-renewal [7,8]. Lung cancer remains one of the leading causes of cancer-related mortality worldwide; it requires a deeper understanding of its molecular underpinnings to develop more effective therapeutic strategies. Among the various effectors contributing to oncogenesis, CBX3 has emerged as a significant player that regulates gene expression and chromatin organization. The intricate molecular structure of CBX3 facilitates its interactions with chromatin, thereby influencing critical cellular processes such as proliferation, differentiation, and apoptosis. Recent studies have highlighted the dysregulation of CBX3 expression in lung cancer tissues compared to adjacent normal tissues, suggesting its potential role as a biomarker for prognosis and treatment response. Various methodologies, including quantitative PCR and immunohistochemistry, have been employed to measure CBX3 expression levels, revealing a compelling correlation between elevated CBX3 levels and poor prognosis in lung cancer patients. In non-small cell lung cancer (NSCLC), CBX3 has emerged as a promising prognostic biomarker. Higher CBX3 expression levels have been linked to the control of cell cycle progression and its potential impact on the PI3K/AKT and Ras signaling pathways [9,10]. However, the precise mechanisms by which CBX3 contributes to the development and progression of lung cancer, including its involvement in different signaling pathways, have not been thoroughly examined and require additional research. Currently, the literature does not provide sufficient information to fully understand the involvement of CBX3 in these diseases, underlining the need to develop more elaborate and precise studies to obtain a greater insight into the function of CBX3 in molecular pathways. Addressing the mechanisms by which CBX3 contributes to lung cancer progression, particularly its involvement in cell proliferation, apoptosis regulation, and the modulation of key oncogenic pathways, will open novel avenues for therapeutic intervention. Current strategies aimed at inhibiting CBX3 activity in lung cancer are being explored. They showed promising results observed in preclinical models; however, concerns regarding potential side effects necessitate careful consideration. As research continues to uncover the multifaceted roles of CBX3 in lung cancer, there is a growing interest in its integration into personalized medicine approaches and the identification of challenges and opportunities for the therapeutic targeting of CBX3. This review aims to give a detailed overview of the therapeutic potential of CBX3. We analyze the main upstream regulators of CBX3 expression and activity and describe the various pathways involving CBX3, highlighting its differential mechanisms of action as well as its significance as a potential therapeutic biomarker in lung cancer.

## 2. CBX3/HP1γ Protein in Cancer Proliferation

**Figure 1:**
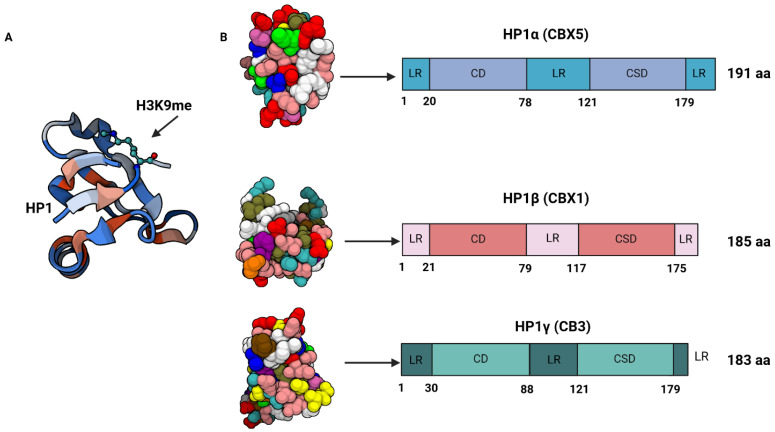
(**A**). Ribbon diagram showing chromodomain of HP1 complexed with histone H3 tail containing monomethyl lysine 9. (**B**). Crystal structure of HP1α, HP1β, HP1γ chromoshadow domains (left); schematic representation of HP1 isoform proteins (right) [23]. LR = linker region; CD = chromodomain; CSD = chromoshadow domain.

The HP1 family in mammals consists of three distinct yet remarkably conserved non-histone homologs, namely CBX1/HP1β, CBX3/HP1γ, and CBX5/HP1α [11,12]. The chromodomain proteins of HP1 and the polycomb group (PcG) exhibit a significant degree of amino acid sequence similarity, with over 60% identity [13]. The criticality of the CHD of HP1 lies in its association with chromatin, which is facilitated by the specific interaction between the CHD and histone H3 lysine K9 di/trimethylation (H3K9me2/3). The strength of the binding affinity between the CHD and H3K9me2/3 was found to be directly proportional to the higher levels of H3K9me2/3 [14,15]. The carboxyl-terminal region of the HP1 protein family contains a second conserved domain known as the chromoshadow domain (CSD) (Figure 1) [16]. Although the general architecture of the CSD resembles that of the CHD, these domains exhibit distinct functionalities. The CSD functions primarily as a dimeric domain; therefore, HP1 proteins readily form homodimers and heterodimers via their CSDs [15,17,18]. With regard to CBX3, its principal function is the establishment of heterochromatin, which represents the condensed state of chromatin. Within the chromatin structure, the “co-packed state” corresponding to heterochromatin is associated with gene transcriptional inactivation and/or gene silencing. Transcriptional inactivation is mediated by the binding of the CBX3 protein to regions of DNA that have undergone methylation at histone H3 lysine K9 (H3K9) via a positive feedback loop [19]. CBX3 can recognize and bind the H3K9me2 and H3K9me3 marks. Subsequently, these modifications facilitate the recruitment of the H3K9 methyltransferase known as histone–lysine N-methyltransferase SUV39H1 to methylate neighboring H3K9 residues [14]. The diffusion of H3K9me3 marks is concomitant with the recruitment of multiple proteins, which elicit chromatin compaction and transcriptional repression by sequestering genes, rendering them transcriptionally inactive [20,21,22].

CBX3, rendered as a soluble nuclear protein and HP1 family member, is encoded by the *CBX3* gene and is localized on chromosome 7p15.2 [24]. In addition, at the subcellular level, it is localized to the nucleoplasm and nuclear bodies. CBX3 links methylation marks to RNA splicing, DNA repair, and transcriptional silencing resulting in them being involved in various cellular processes, such as gene regulation, DNA repair, and telomere function [25,26]. Importantly CBX3 is regarded as a multifaceted crystal-structured protein in humans that also has a function in transcriptional inhibition and activation, cell growth and differentiation, and epigenetic modifications [27,28]. The CBX3 chromodomain recognizes and binds with non-histone and histone-methylated peptides and based on comparable affinities, also binds with H1K26-, H3K9-, and G9aK185-methylated peptides [19]. The binding of the CBX3 chromodomain to methylated histones occurs via a conserved mechanism, which is enhanced by the chromodomain ARKS/T motif, allowing for the chromodomain to recognize and specifically bind to methylated histones [26,29]. CBX3 also interacts with non-histone proteins, including PIM1, CBX5, CBX1, Ki-67, and Lamin B receptor, controlling gene expression [26]. CBX3 interacts directly with active genes, particularly within gene bodies, and facilitates the process of transcriptional elongation and RNA processing. It is also involved in recruiting splicing factors to enable efficient co-transcriptional splicing [30]. CBX3 interacts with the E2F1 transcription factor, a key player in regulating the cell cycle. Several studies report that cellular proliferation is enhanced by the increase in E2F1 transcriptional activity mediated by CBX3. E2F1 selectively directs its binding toward genes that encode proteins responsible for the regulation of cell cycle progression during the transition from the G1 to the S phase, including cyclin D1 and CDK4 [31,32]. CBX3 can interact with the tumor suppressor protein p53, impeding its transcriptional activity and resulting in apoptosis reduction and a concomitant increase in cell survival. The primary role of p53 under cellular stress, such as oncogenic activation and DNA damage, is to induce apoptosis regulated by pro-apoptotic genes such as BCL2-associated X, Apoptosis Regulator (*BAX*), and phorbol-12-myristate-13-acetate-induced protein 1 (*PMAIP1*, also known as *NOXA*), as well as the transcriptional activation of the p53 upregulated modulator of apoptosis (PUMA) [33]. CBX3 was found to be able to indirectly enhance the transcriptional activation of genes involved in DNA repair, including *RAD51* and breast cancer gene 1 (*BRCA1*). *RAD51* plays a critical role in repairing DNA double-strand breaks (DSBs) via homologous recombination (HR) [34,35]. A recent study described an association between mutations in the BRCA1 protein, which has a role in HR repair, and a higher susceptibility to breast and ovarian cancers [36]. The interface between CBX3 and E2F1 enhances the expression of *RAD51* and *BRCA1*, resulting in increased HR repair and resistance to chemotherapy. CBX3 also interacts with other DNA repair proteins, such as PARP1 and Ku70, which participate in the repair of DSBs through non-homologous end joining [37]. Through its interactions, CBX3 plays a crucial role in multiple DNA repair pathways either directly or indirectly by recruiting DNA repair proteins, thereby maintaining genomic stability [34,35,36].

## 3. Upstream Regulators of CBX3 Expression and Activity

Many transcription factors are known to regulate the expression of CBX3, highlighting an intricate network of gene expression regulation. For instance, ZEB1 and ZEB2 have been identified as pivotal transcription factors that may influence the transcriptional regulation of CBX3, particularly in tumor cells [26]. This interaction suggests a significant role for CBX3 in oncogenic processes, where it might be part of broader regulatory mechanisms driving tumor progression. In addition, the study of transcription factors and miRNAs associated with CBX3-binding hub genes provides further insights into the molecular mechanisms governing its expression. These findings underscore the complexity of CBX3 transcriptional regulation highlighting multiple factors and pathways converging to modulate its expression. Intriguingly, understanding these interactions can offer valuable perspectives on how CBX3 contributes to cellular differentiation and disease states, thus emphasizing the need for detailed molecular characterizations and targeted interventions in related therapeutic contexts. The interaction between transcription factors and promoter regions is vital for regulating gene expression, as evidenced by the interaction between CBX3 and the NCAPG promoter. Interestingly, luciferase reporter assays have shown that CBX3 enhances the transcriptional activity of the NCAPG promoter in HCT116 cells [38]. Further supporting this, overexpression of CBX3 (Ov-CBX3) resulted in elevated expression levels of NCAPG in the same cell line, emphasizing the regulatory role of CBX3 on the NCAPG promoter [3]. Human TFDB predictions corroborated these findings by identifying binding sites for CBX3 on the NCAPG promoters, suggesting a direct interaction between CBX3 and these promoter regions [38]. Such data elucidate the role of CBX3 in transcriptional regulation, indicating that transcription factors, like CBX3, can modulate gene expression by directly binding promoter regions thus affecting transcriptional activity. This interaction exemplifies the broader principle of transcription factor–promoter interactions in gene regulation, underscoring the need for further studies to explore the mechanistic details and potential therapeutic interventions targeting such pathways. Building on this evidence, further experimental analysis substantiates the involvement of transcription factors in CBX3 regulation. Despite CBX3 binding being widespread across actively transcribed genes, the absence of large-scale transcript alterations upon CBX3 knockdown underscores a nuanced regulatory mechanism. Interestingly, CBX3-bound genes were observed to have significantly higher expression levels than unbound genes in wild-type cells, suggesting that CBX3 binding is preferentially associated with highly expressed genes [26]. These analyses collectively highlight the critical role of CBX3 in gene regulation and the involvement of transcription factors in modulating its activity, emphasizing the need for further studies to elucidate the detailed mechanisms and potential therapeutic interventions targeting CBX3-related pathways. Recent studies have elucidated the complex regulatory mechanisms involving non-coding RNAs that influence CBX3 expression, particularly highlighting the significant role of miR-375. miR-375 has been validated as a direct target for CBX3, suggesting its crucial involvement in colorectal adenocarcinoma (COAD) progression [39]. In this context, the study uncovered that the long non-coding RNA (lncRNA) SNHG17 can modulate COAD progression through its interaction with the miR-375/CBX3 axis, indicating a sophisticated regulatory network [39]. Non-coding RNAs play a crucial role in the regulation of CBX3, particularly in the context of hepatocellular carcinoma (HCC). One of the prominent mechanisms involves the miR-139/CBX3 axis. miR-139 functions as a direct target of CBX3, establishing a feedback loop that influences the progression of HCC by regulating cell cycle progression. Specifically, miR-139 downregulates CBX3, which in turn leads to cell cycle arrest and inhibits the proliferation of HCC cells [40]. This regulatory pathway underscores the potential of targeting miR-139 and CBX3 interactions for therapeutic interventions in HCC. The interactions between non-coding RNAs (ncRNAs) and regulatory proteins have emerged as a critical area of research, particularly concerning their influence on CBX3. Long non-coding RNAs (lncRNAs) are especially noteworthy due to their ability to interact with DNA, proteins, and other RNA molecules through their complex three-dimensional structures [41]. This multifaceted interaction enables lncRNAs to modulate transcriptional regulation and chromatin architecture. For instance, scaffold lncRNAs can assemble protein complexes that influence chromatin states, thereby playing crucial roles in epigenetic regulation [42]. In the context of CBX3, ncRNAs may influence its regulatory functions by acting as molecular decoys for RNA-binding proteins or by altering chromatin dynamics. The lncRNA NORAD exemplifies this mechanism by serving as a decoy for RNA-binding proteins, which could similarly affect CBX3-related pathways [43]. Moreover, the interaction between nascent RNA and chromatin can link transcriptional output with chromatin modifications, which might manifest in the regulation of CBX3 and its target genes [44]. Taken together, these findings indicate that ncRNAs are not merely passive elements but active participants in the regulatory networks involving CBX3. Epigenetic modifications are also crucial for the regulation of CBX3. Among the various types of epigenetic regulation, DNA methylation and histone modifications are prominently associated with CBX3 activity. DNA methylation involves the addition of a methyl group to the DNA molecule, typically at cytosine bases, which can lead to long-term gene silencing and is a stable, heritable change that does not alter the nucleotide sequence itself [45,46]. Histone modifications, such as methylation, acetylation, and phosphorylation, alter the chromatin structure and influence the accessibility of DNA to transcriptional machinery. Specifically, histone methylation is known to affect transcriptional regulation by recruiting transcriptional repressors or activators and modifying chromatin architecture [29]. These modifications can directly impact the binding of regulatory proteins, such as methyl-CpG-binding proteins, transcription factors, and RNA molecules, thus ensuring precise control over gene expression [14]. Understanding these epigenetic mechanisms is essential for comprehending how CBX3 contributes to gene regulation and the broader implications for cellular function and disease states. Consequently, these findings underscore the need for further investigation into the specific regulatory pathways mediated by CBX3 to unveil its full spectrum of biological functions.

## 4. CBX3 as a Multiplayer in Lung Cancer Progression

CBX3 has been found to be dysregulated showing an abnormal expression profile in various cancers. Expression levels of this gene are increased in several cancer types including gastric, prostate, breast, colorectal, and lung cancers. CBX3 expression is also dysregulated in osteosarcoma and hepatocellular carcinoma [27,37,47,48]. Conversely, expression levels of CBX3 are lower in colorectal cancer low-grade adenomas and hyperplastic and mucosal polyps [37]. Among the three HP1 proteins, CBX3 is the histone reader protein that is highly expressed in lung adenocarcinoma (LUAD). The expression level of messenger RNA (mRNA) encoding CBX3 exhibits a positive correlation with the size of tumors, the occurrence of lymph node metastasis, and unfavorable prognosis in LUAD patients. Interestingly, the in vivo inhibition of CBX3 results in a reduction in tumor size and an extension of the survival period in mice with KRAS^G12D^-induced LUAD [49]. Increased expression of CBX3 is correlated with an unfavorable prognosis in NSCLC and LUAD [50,51] through mechanisms involving the promotion of tumor proliferation via regulatory pathways of signal transduction affecting the cell cycle, notably G1/S phase transition and the p53 pathway [10]. The prognostic value of CBX3 is further supported by its association with tumor diameter and lymph node metastasis, suggesting its involvement in tumor growth and metastasis [10]. The therapeutic potential of targeting CBX3 in lung cancer is underscored by its overexpression in NSCLC and its association with epigenetic modifications and cell differentiation [27]. The oncogenic role of CBX3 is also highlighted by the observation that CBX3 and H3K9me3 levels are increased in NSCLC tumor-initiating cells, where they inhibit DNA damage responses to antineoplastic agents [50]. The expression of CBX3 is markedly increased in LUAD tissues of smokers compared to non-smokers and it is also associated with unfavorable prognosis and advanced disease stage [52]. Interestingly, a study shows that cigarette smoke causes an increase in CBX3 expression by promoting the binding of the transcription factor YBX1 to the CBX3 promoter [27]. High CBX3 protein levels also enhance the growth, invasion, and spread of LUAD cells by controlling the cell cycle progression and activating Rho GTPases [53]. Notably, an elevated expression of CBX3 in lung cancers linked to smoking is often caused by genetic changes, such as an increase in the number of copies of the gene, as well as in epigenetic dysregulation [54]. In smokers, it has also been observed that CBX3 interacts with tripartite motif-containing (TRIM) 28, TRIM24, and RBBP4 to create a repressor complex. This complex binds to the Rho GTPase-activating protein 24 (ARHGAP24) promoter and inhibits its transcription. Reducing levels of ARHGAP24 results in the overexpression of active Ras-related C3 botulinum toxin substrate 1 (RAC1) that, in turn, triggers signaling pathways (see also Section 5.6) promoting the advancement of LUAD. Outside the CBX3/ARHGAP24/RAC1 axis, CBX3 can also facilitate smoking-induced LUAD by inhibiting the tumor suppressor FBP1 and controlling glycolysis [52]. Intriguingly, CBX3 can also enhance the development of lung tumors by suppressing the transcriptional activity of nuclear receptor co-repressor 2 (NCOR2) and zinc finger and BTB domain-containing 7A (ZBTB7A). These transcriptional regulators have an impact on cell proliferation and migration [55]. Further, the expression of CBX3 triggers the development of stem cell-like characteristics in lung tumors, enhancing the presence of markers associated with cancer stem cells and targets of the oncogenic transcription factor c-Myc [56]. Mechanistically, CBX3 plays a crucial role in suppressing target genes through chromatin remodeling, leading to abnormal cell development and the inhibition of differentiation pathways [27,56]. Due to its cancer-causing properties, CBX3 shows potential as a reliable predictive biomarker and a possible target for treatment in NSCLC associated with smoking. Manipulating the expression or activity of CBX3 could potentially limit the development of lung tumors and improve patient prognosis. Studies suggest that exposure to cigarette smoke can lead to specific alterations in the histone organization of lung cells and that these changes can affect how transcription factors bind to promoters of genes, including CBX3 [52,57]. Although there is no direct evidence to support this hypothesis, it is suggested that the transcription factor NF-κB, known to be activated by cigarette smoke, can potentially regulate the expression of target genes such as CBX3 [58,59].

The different interactors of CBX3, their molecular mechanisms, and the different pathways involved in lung cancer are listed in Table 1.

## 5. Involvement of CBX3 in Pathways Leading to Lung Cancer

**Figure 2:**
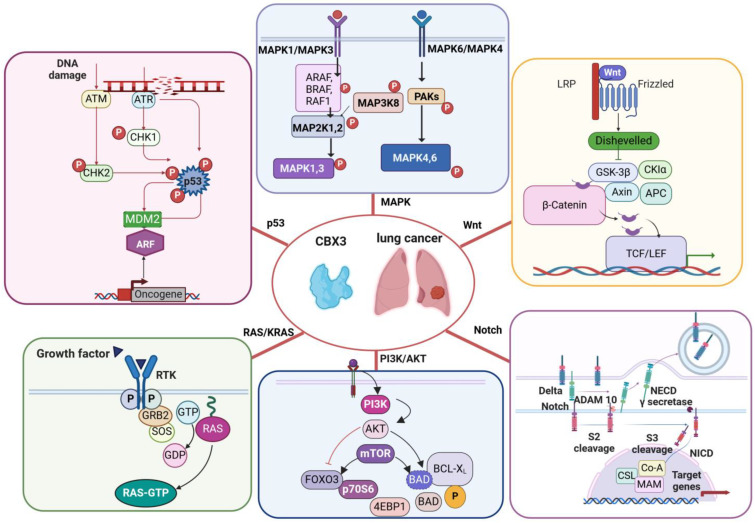
Cellular pathways directly or indirectly modulated by CBX3 in lung cancer.

Molecular pathways involved in the regulation of the cell cycle, differentiation, death, and signaling are known to be altered in processes of tumorigenesis. To date, major efforts have been made to discover new cancer driver genes and unravel the molecular mechanisms in which they are involved by combining scientific data, such as multi-omics data, and knowledge obtained from the literature. For example, therapies based on molecular targets have transformed anticancer treatment approaches through personalized and/or precision medicine strategies. Based on this premise, CBX3 has been found implicated in a broad spectrum of human cancers, including NSCLC [10,60]. Remarkably, CBX3 is involved in several signaling pathways, critical for cell survival, proliferation, and differentiation, and plays a dominant role in lung cancer. However, further research is needed to better elucidate these pathways and explore the potential of CBX3 as a therapeutic target in depth. The following subsections describe the role of CBX3 in key cancer-associated pathways and aim to shed light on its mechanistic role in lung cancer progression. The involvement of CBX3 in crucial lung cancer networks is schematically illustrated in Figure 2.

### 5.1. Role of CBX3 in PI3K-AKT and (K)Ras Signaling Pathways

Recent studies describe a significant connection between CBX3 and the activation of the PI3K-AKT pathway. This pathway regulates essential cellular processes such as growth, survival, and metabolism [62]. The aberrant activation of the PI3K-AKT pathway is a well-known characteristic of tumor development. In this context, CBX3 was found to play a role in PI3K-AKT dysregulation by facilitating the phosphorylation and subsequent activation of AKT. CBX3 may act as an oncogenic driver and is potentially involved in the Ras signaling pathway, one of the most crucial molecular mechanisms inducing oncogenic transformation. Enrichment analysis seems to support this hypothesis, but further research is needed to establish the specific role of CBX3 in this pathway in lung cancer [65]. A functional relationship is known to exist between CBX3 and EGFR or RAC1 in different human cancers [60], potentially impacting these signaling pathways. CBX3 has a significant effect on the KRAS signaling pathway in lung cancer. Specifically, CBX3 preferentially interacts with EZH2 triggering the transcription inhibition of microRNAs (miRNAs) such as let-7b, miR-31, and miR-128b. This results in the upregulation of target genes, including *KRAS* and *MYC*, that stimulate the growth and survival of tumor cells [66]. CBX3 also promotes oncogenic KRAS signaling, activating downstream effector pathways such as MAPK/ERK and PI3K/AKT. This activation enhances cancer cell proliferation, invasion, and metastasis [67]. Of note, a strong association is known to exist between elevated CBX3 levels and unfavorable prognosis in individuals with LUAD harboring KRAS mutations [68]. The Hippo pathway, which regulates organ size and suppresses tumors, often becomes dysregulated in NSCLC cells harboring KRAS mutations, particularly the KRAS G12C variant [69]. Interestingly, an association exists with the activation of PI3K/AKT signaling as one of the survival signals [70]. There is plenty of evidence for glutamine’s essential role in tumors, and, as a variety of other factors, the tissue type, underlying cancer genetics, tumor microenvironment, and other independent variables such as diet and host physiology together but not uniformly influence the role of glutamine in cancer. Therefore, requirements for glutamine in cancer are overall very heterogeneous [71]. Recent studies demonstrated that CBX3 was involved in regulating an interaction network consisting of different genes which may subsequently participate in glutathione metabolism. In addition, CBX3 also exhibited a negative correlation with glycosphingolipid metabolism, which may be associated with the regulation of CBX3 on DNA methylation [72]. Another recent study exploited a bypass of a late G1 glutamine (Gln)-dependent cell cycle checkpoint in cancer cells with KRAS mutations. Upon Gln deprivation, KRAS-driven cancer cells enter the S phase and arrest due to insufficient nucleotide biosynthesis. The S-phase arrested cells are then vulnerable to the cytotoxic drugs capecitabine, paclitaxel, and rapamycin. Thus, Gln deprivation creates a “synthetic lethality” for capecitabine, paclitaxel, and rapamycin in KRAS-driven cancer cells [73]. These findings highlight the potential of CBX3 as both a prognostic biomarker and a therapeutic target. Its association with these critical oncogenic signaling pathways further supports the importance of gaining a better insight into its role in cancer progression [61]. It has been observed that CBX3 promotes lung tumorigenesis by interacting with cancer-specific pathways, leading to increased cell proliferation and survival. Evidence suggests that smoking-associated upregulation of CBX3 can accelerate the progression of lung adenocarcinoma (LUAD) by activating the ARHGAP24/Rac1 signaling axis [74]. Combining CBX3 inhibitors or modulators with existing cancer therapies could potentially enhance therapeutic efficacy. Moreover, building on the established association between CBX3 and the PI3K/AKT pathway, CRISPR/Cas9 technology offers a promising route to manipulate CBX3 activity and elucidate its role in cellular processes. The identification of CBX3 as a biomarker for lung cancer, combined with its significant association with immunity, reinforces its potential as a viable therapeutic target within the PI3K-AKT pathway. The frequent co-occurrence of KRAS mutations and CBX3 expression underscores a potential synergistic effect in driving tumorigenesis. Understanding the exact molecular mechanisms through which KRAS mutations influence CBX3 function, and vice versa, could open new avenues for targeted therapies in lung cancer patients harboring these mutations.

### 5.2. Role of CBX3 in Notch Signaling Pathway

In the context of lung cancer, the involvement of CBX3 in the Notch signaling pathway is intricate and diverse, underscoring the complex interplay between chromatin organization and signaling pathways in cancer progression [75]. The Notch signaling pathway is a crucial cell communication system in determining cell fate. CBX3 is able to interfere with the functioning of this pathway, and its effect may vary depending on the specific cellular environment and cancer type [76]. The Notch signaling pathway is highly conserved and involves the interaction of Notch receptors with their ligands, leading to cleavage of the Notch intracellular domain (NICD) and its translocation to the nucleus, where it influences gene expression [77,78]. However, the involvement of CBX3 in lung cancer, and particularly its relationship with the Notch signaling pathway, remains relatively understudied and seems to present a more complex scenario. CBX3 directly interacts with the NICD, recruiting the co-repressor complex, including histone deacetylases and DNA methyltransferases, to the promoter regions of Notch3 target genes, such as *HES1* and *HEY1* [79]. This interaction leads to the epigenetic silencing of these genes through increased histone deacetylation and DNA methylation, ultimately resulting in the downregulation of the entire signaling pathway [79]. Thus, chromatin remodeling may arise due to the ability of CBX3 to modify histones and change the chromatin state, consequently affecting the accessibility of Notch-responsive elements in the genome. In addition, since CBX3 has been linked to epigenetic changes such as H3K9me3, which plays a role in regulating the response to DNA damage, we speculate that it could potentially contribute to the resistance of tumor-initiating cells in NSCLC to antineoplastic drugs. This specific resistance is potentially conferred due to CBX3 binding to H3K9me3, resulting in the formation of transcriptionally repressive chromatin environments that can lead to the silencing of tumor suppressor genes in drug metabolism and efflux, contributing to drug resistance [50,80]. Interestingly, CBX3 expression has also been correlated to immune-related function regulation, also regulated by Notch signaling [81]. These interactions could additionally modulate the tumor microenvironment, supporting tumor growth as well as resistance to various therapies. In the case of NSCLC, CBX3 might indirectly favor drug resistance by influencing immune evasion and immune cell infiltration mechanisms [61]. Building on the evidence of CBX3 overexpression in lung cancer tissues, the integration of Notch signaling pathway inhibitors with CBX3 targeting represents a promising strategy for therapeutic intervention. The Notch signaling pathway plays a crucial role in regulating cancer stem cells and mediates resistance to conventional therapies, making it a critical target for on-target therapeutic strategies [82]. By inhibiting the Notch pathway, one can potentially disrupt the self-renewal and survival mechanisms of cancer stem cells, thereby enhancing the efficacy of CBX3 targeting. Therefore, combining Notch signaling inhibitors with CBX3 targeting could enhance therapeutic outcomes by simultaneously attacking the cancer from multiple angles. This dual-targeting strategy could potentially overcome the resistance mechanisms often encountered in cancer therapy, providing a more comprehensive and effective treatment approach. 

### 5.3. Role of CBX3 in Wnt Pathway

CBX3 regulates the Wnt/β-catenin signaling pathway, essential for cell proliferation, differentiation, and tumorigenesis in several types of cancer, including lung cancer [83]. The mode of action of CBX3 in lung cancer involves its function as a transcriptional regulator. CBX3 is able to bind gene promoters and influence gene expression. A recent study showed that CBX3 plays a crucial role in the transcriptional regulation of the non-structural maintenance of chromosomes’ condensin I complex subunit G (*NCAPG*). This regulation, in turn, leads to activation of the Wnt/β-catenin signaling pathway. In colorectal cancer, activation of this pathway promotes cell proliferation and cell cycle progression, while inhibiting apoptosis. This mechanism likely operates in a similar manner in lung cancer, as the pathways involved in tumor formation are shared [38]. CBX3 overexpression has been linked to the advancement of lung adenocarcinoma via activation of the RAC1 pathway, a component of the Wnt signaling network [60,61]. Previous studies found a connection between CBX3 and cell cycle control [84]. Specifically, CBX3 was found to reduce the transition from the G1 to the S phase by influencing the activity of p21 [25,53]. This, in turn, may contribute to the growth of tumors. It is also reported that CBX3 might have an impact on tumor growth by engaging with cell cycle regulators [25,85]. To summarize, CBX3 functions as a transcriptional regulator in the Wnt/β-catenin signaling pathway, impacting the advancement of lung cancer by controlling gene expression, facilitating cell cycle progression, and interacting with pathways such as RAC1. In this context, the overexpression of the CBX3 gene is associated with a negative outlook, suggesting its potential role as a target for treatment and a useful indicator of lung cancer prognosis [38,53]. One of the most promising strategies involves the inhibition of CBX3 to enhance the efficacy of existing Wnt-targeted therapies. By downregulating CBX3, the repressive effect on Wnt signaling may be alleviated, allowing for more effective activation of Wnt-related therapeutic agents. Furthermore, the development of small molecules specifically designed to inhibit CBX3 could represent a breakthrough in lung cancer treatment. These small molecules would potentially offer a new class of cancer therapies that selectively target the dysregulated mechanisms at play in lung tumors, rather than broadly affecting all rapidly dividing cells. Additionally, targeting CBX3 holds the potential for personalized therapy options for lung cancer patients who exhibit specific mutations impacting the Wnt pathway. By tailoring treatments based on individual patient profiles, therapeutic efficacy could be enhanced while minimizing adverse effects associated with more generalized treatments.

### 5.4. Role of CBX3 in p53 Pathway

The tumor suppressor p53, a transcription factor responsible for initiating cell cycle arrest, apoptosis, and DNA repair when cells undergo stress, is involved in the multiple mechanisms that provide evidence supporting the role of CBX3 in lung cancer progression [86]. The p53 activity is typically suppressed in normal cells by specific degradation regulated by the E3 ubiquitin ligase mouse double minute 2 homolog (MDM2) [86]. MDM2 primarily targets p53 via proteasomal degradation. In addition, its auto-ubiquitination activity does not directly affect MDM2 itself, but rather its interactions with other proteins [87]. It has been observed that CBX3 interacts with and potentially enhances the stability of MDM2 in lung cancer cells, inhibiting its auto-ubiquitination. However, the precise mechanism by which CBX3 enhances the stability of MDM2 is still unknown, and more research is required to elucidate this interaction [27,87]. CBX3 facilitates the survival and growth of lung cancer cells by indirectly impeding the activity of p53 through the MDM2 axis [88]. Additionally, CBX3 can directly suppress the expression of p53 target genes responsible for inhibiting growth, regardless of its impact on p53 protein levels [89]. Collectively, these findings show that CBX3 is able to bypass the tumor-suppressing effects of p53 and promote the development of lung tumors [90]. Thus, targeting the CBX3-MDM2-p53 pathway could potentially offer a novel therapeutic approach to restore the functionality of p53 in lung cancer. Furthermore, the intricate relationship between CBX3 and p53 highlights the potential for developing therapies that exploit this interaction to reactivate p53’s tumor suppressor functions. Given that mutant forms of p53 often exhibit oncogenic properties that facilitate cancer cell proliferation and survival, strategies aimed at modulating CBX3 could restore the normal function of p53, thereby reinforcing its role as a critical tumor suppressor. Thus, the modulation of CBX3 represents a promising approach for enhancing the efficacy of p53-based cancer therapies, necessitating further research to fully understand the therapeutic potential and underlying mechanisms.

### 5.5. Role of CBX3 in ErbB Pathway

The ErbB signaling pathway is crucial in controlling numerous cellular processes, such as differentiation, proliferation, migration, adhesion, and apoptosis [91]. This pathway is activated by EGF-like growth factor ligands’ attachment to the outer part of ErbB receptors [92]. This causes the receptors to form dimers, which can be either homodimers or heterodimers with other members of the same family. The ErbB family consists of four members: ErbB1 (HER1), ErbB2 (HER2), ErbB3 (HER3), and ErbB4 (HER4) [93]. When the receptors dimerize, their inherent tyrosine kinase activity is triggered, leading to autophosphorylation and activating the downstream signaling cascades, including the MAPK, Akt, and JAK/STAT pathways [92,93]. Although the precise mechanism by which CBX3 contributes to lung cancer is far from being completely elucidated, some evidence points to its potential interaction with EGFR signaling. Intriguingly, a co-occurrence of positive CBX3 expression and EGFR mutations was found in NSCLC samples [51]. However, in the same study, the expression of CBX3 remained unaltered in EGFR mutant NSCLC cell lines treated with the EGFR inhibitor gefitinib [94], suggesting that the downstream signaling of EGFR may not influence the expression of CBX3 [91]. CBX3 is overexpressed in NSCLC and is associated with an unfavorable prognosis. Nonetheless, further investigation is required to clarify its exact function in interacting with the ErbB/EGFR pathway during the advancement of lung cancer. Specifically, future studies should focus on determining whether CBX3 governs the expression of ErbB pathway members via epigenetic regulation, investigating the impact of EGFR signaling on CBX3 activity, and elucidating the mechanisms by which CBX3-mediated gene regulation enhances lung cancer development and survival. The role of CBX3 in lung cancer progression is intricate and multifaceted, particularly in its interactions with ErbB receptors. Specifically, the overexpression and overactivation of ErbB receptors, which are known to correlate with poor prognosis, drug resistance, cancer metastasis, and lower survival rates, highlight a pivotal area of concern in lung cancer research [95]. Examining the relationship between CBX3 and ErbB receptors would be useful to uncover potential therapeutic targets to mitigate the aggressiveness of lung cancer. Given the complex interplay between these proteins and pathways, further studies are necessary to elucidate the detailed mechanisms and develop effective interventions.

### 5.6. Role of CBX3 in MAPK Pathway

The MAPK/ERK pathway is a crucial signaling cascade that regulates cellular growth and survival [52]. CBX3 can inhibit transcription of the *ARHGAP24* gene by interacting with H3K9me3 and binding to proteins that modify the structure of chromatin, such as TRIM28 and TRIM24, at the *ARHGAP24* promoter. As a result, the expression of *ARHGAP24* is suppressed, leading to an increase in RAC1 activity. Subsequently, RAC1 stimulates receptor tyrosine kinases on the cell membrane, propagating signals through the conventional MAPK cascade involving MEK1/2, ERK1/2, Ras, and Raf. Ultimately, activated ERK1/2 migrates to the nucleus and phosphorylates many transcription factors that control the proliferation and survival of cancer cells [96]. CBX3 also impacts other pathways such as PI3K/AKT and Ras signaling, which converge on MAPK. For example, AKT is able to phosphorylate and inhibit Raf proteins, providing crosstalk between these pathways. Ras functions directly upstream of Raf in the MAPK cascade [96]. Inhibiting CBX3 could have profound implications for the MAPK pathway in lung cancer cells, primarily due to the intricate network of interactions that CBX3 maintains with various signaling pathways. Specifically, the CBX3-MAPK axis represents a potential therapeutic target, as the dysregulation of both CBX3 and MAPK pathways significantly contributes to tumor growth and survival. This interconnection suggests that targeting CBX3 could disrupt the MAPK signaling cascade, potentially leading to reduced tumor proliferation and enhanced cancer cell apoptosis. Thus, interventions aimed at inhibiting CBX3 could not only attenuate MAPK pathway hyperactivation but also impede the broader oncogenic network, providing a multifaceted approach to lung cancer therapy. This highlights the necessity for further research into CBX3 inhibitors as a means to develop more effective treatments for lung cancer.

## 6. Conclusions

Lung cancer is one of the leading causes of cancer-related deaths in industrialized countries due to extremely variable contributing factors such as detrimental lifestyle behaviors, including smoking [97]. Therefore, the aberration of epigenetic mechanisms significantly impacts gene expression and diverts cellular signaling pathways that are crucial for coordinating the correct growth, regulation, and functioning of cells. In lung cancer, and especially in NSCLC, CBX3 appears to act primarily as an oncoprotein, promoting tumor growth and progression. Multiple studies show that CBX3 is overexpressed in NSCLC [51] and correlates with poor prognosis [10]. Mechanistically, CBX3 interacts with key oncogenic pathways such as PI3K/AKT, Ras, Wnt/β-catenin, and p53, thereby stimulating proliferation, inhibiting apoptosis, and increasing therapeutic resistance [76]. It may also contribute to a stem cell-like phenotype in lung cancer cells [56]. The upregulation of CBX3 in smoking-associated LUAD and its inhibition of tumor suppressors such as ARHGAP24 and NCOR2/ZBTB7A provide further evidence for its oncogenic role [52]. 

Targeting CBX3 presents unique challenges, not only due to its essential role in normal cellular functions but also because of the complexity of its interactions within the cellular milieu. Recent studies underlined the mechanisms through which CBX3 inhibitors can exert their effects, including the modulation of specific biochemical pathways that govern cancer cell proliferation. The ongoing development of CBX3 inhibitors has garnered attention, with several promising candidates currently undergoing preclinical and clinical trials, though concerns regarding their potential side effects remain a critical aspect of this research. Furthermore, the exploration of combination therapies involving CBX3 inhibitors is gaining momentum, as these strategies may enhance therapeutic efficacy by synergizing with established cancer treatments. Nevertheless, the path toward effective combination therapies is fraught with challenges, including the need for precise patient selection and the integration of personalized medicine approaches, which could optimize outcomes. As this field of research evolves, emerging trends and potential biomarkers for identifying patients most likely to benefit from CBX3 inhibitors are becoming increasingly relevant, paving the way for innovative treatment paradigms that could significantly impact cancer care. Current strategies for developing CBX3 inhibitors have garnered significant attention due to their potential in addressing drug resistance and improving cancer treatment outcomes. One promising approach involves the use of immune checkpoint inhibitors (ICIs) and cyclin-dependent kinase (CDK) inhibitors to prevent CBX3-mediated tumorigenesis. These inhibitors are proposed as molecularly targeted therapeutics, aiming to disrupt the pathways through which CBX3 contributes to cancer progression [98]. Additionally, the relationship between CBX3 expression and resistance to PI3K or mTOR inhibitors highlights the necessity of developing targeted therapies for patients exhibiting high CBX3 levels. This resistance indicates that CBX3 may play a pivotal role in mediating treatment efficacy and underscores the importance of personalized therapy in combating cancer [10]. These strategies collectively emphasize the need for innovative drug development efforts focused on CBX3, aiming to enhance treatment specificity and effectiveness while overcoming resistance barriers in cancer therapy. Combining CBX3 inhibitors with other therapeutic agents represents a multipronged strategy to enhance the efficacy of cancer treatments, including lung cancer. The known interactions of CBX3 with metabolic pathways further underscore its importance in cellular metabolism. Recent research has revealed that CBX3 is intricately linked with glutamine metabolism, a vital process for rapidly proliferating cells, including cancer cells. Genetic manipulation techniques have proven essential in studying the role of CBX3 in glutamine metabolism. For instance, CRISPR-Cas9 can be used to create knockout models where the CBX3 gene is completely inactivated, providing insights into its metabolic functions by comparing these models to wild-type controls. Additionally, overexpression systems can be employed to study the effects of increased CBX3 levels on cell metabolism. Biochemical assays for metabolic activity are indispensable tools for understanding how CBX3 influences glutamine metabolism. For example, metabolic flux analysis can reveal how the presence or absence of CBX3 affects the rate at which cells convert glutamine into other metabolites. Techniques such as mass spectrometry and NMR spectroscopy provide detailed profiles of cellular metabolites, offering a comprehensive view of metabolic changes. Given the pivotal role of CBX3 in cancer metabolism, targeting CBX3-related pathways presents a promising therapeutic strategy. Targeting each step of glutamine metabolism has shown promising results in cancer treatment, prompting the discovery of druggable targets and novel therapeutic approaches [71,72,73]. Future research should focus on delineating the regulatory networks involving CBX3 and exploring the therapeutic implications of targeting its interactions to restore apoptotic pathways and inhibit cancer cell proliferation. Additionally, understanding the structural nuances of CBX3 and its transcript variants can provide deeper insights into its regulatory complexity and functional diversity, thereby paving the way for innovative strategies in lung cancer therapy.

## Figures and Tables

**Table 1:** CBX3 interactors, their effects on lung cancer, and pathway involvement via differential mechanisms of action.

Interactor	Type of Interaction	Effect on Lung Cancer	Pathway Involvement	Mechanism of Action
EGFR/RAC1	Genetic Interaction	Co-amplification with CBX3 is associated with lung adenocarcinoma proliferation and poor prognosis	Not Specified	Increase in CBX3 mRNA leads to increased EGFR/RAC1 protein levels, promoting cancer cell proliferation [60]
CDK6/P21	Transcriptional Regulation	CBX3 inhibits transcription of negative cell cycle regulators, promoting colorectal cancer cell proliferation; similar mechanisms may be involved in lung cancer	Cell Cycle Regulation	CBX3 is able to inhibit transcription of CDK6 and p21, promoting cell proliferation [61]
CBX Molecular Family (CBX1/2/3/5/7)	Gene Expression	CBX3/5 expression is associated with poor prognosis in lung adenocarcinoma, while CBX7 shows the opposite effect	Tumorigenesis and Immune Infiltration	Differential expression of CBX family members affects tumor progression and immune response [54]
PI3K/AKT Pathway	Activation	Although the study is on renal carcinoma, similar activation by CBX3 may occur in lung cancer, promoting metastasis and invasion	PI3K/AKT Pathway	CBX3 promotes cancer progression through PI3K/AKT activation, which regulates cell metastasis and invasion [62]
ARHGAP24	Suppression	Smoking-associated upregulation of CBX3 suppresses ARHGAP24, activating RAC1 signaling and promoting tumor progression in lung adenocarcinoma	RAC1 Signaling	CBX3 overexpression leads to suppression of ARHGAP24, activating RAC1 and promoting tumor progression [52]
NCOR2	Regulation	In ovarian cancer, CBX3 inhibits NCOR2, affecting p53/p21-mediated glucose metabolism; similar effects may occur in lung cancer	Glucose Metabolism	CBX3 inhibits NCOR2, affecting p53/p21-mediated pathways and potentially promoting cancer metabolism [63]
Immune System	Immunological Biomarker	CBX3 expression is related to immune cell infiltration and may serve as an immunological and prognostic biomarker in various cancers, including lung cancer	Immune Response	CBX3 expression influences immune cell infiltration and tumor immunity, which varies based on tumor type [61]
Transcriptome/Metabolome	Biomarker Association	Possible association between CBX3 expression and transcriptome/metabolome changes in cancers, including lung cancer	Various Pathways	CBX3 expression may be linked to changes in the transcriptome and metabolome, affecting multiple cancer-related pathways [64]

## Data Availability

Data sharing is not applicable.

## Abbreviations

ARHGAP24	Rho GTPase-activating protein 24
BAX	BCL2-associated X protein
BRCA1	breast cancer gene 1
CBX	chromobox
CHD	chromodomain
COAD	colorectal adenocarcinoma
CSD	chromoshadow domain
DSBs	DNA double-strand breaks
DSBs	double-strand breaks
EGFR	epidermal growth factor receptor
EZH1/2	enhancer of zeste homologous ½
HCC	hepatocellular carcinoma
HR	homologous recombination
lncRNAs	long non-coding RNAs
LUAD	lung adenocarcinoma
MDM2	mouse double minute 2 homolog
mRNA	messenger RNA
SMC	structural maintenance of chromosomes
ncRNAs	non-coding RNAs
NCAPG	non-SMC condensin I complex subunit G
NICD	Notch intracellular domain
NSCLC	non-small cell lung cancer
PI3K	phosphoinositide 3-kinase
PMAIP1	phorbol-12-myristate-13-acetate-induced protein 1
PRC1	polycomb (Pc) inhibitory complex 1
PRC2	polycomb repressive complex 2
PUMA	p53 upregulated modulator of apoptosis
RAC1	active Ras-related C3 botulinum toxin substrate 1
RBBP4/7	retinoblastoma binding protein 4/7
STAT3	signal transducer and activator of transcription 3
TRIM	tripartite motif-containing
ZBTB7A	zinc finger and BTB domain-containing 7A

## References

[1] Tang Q., Cheng J., Cao X., Surowy H., Burwinkel B. (2016). Blood-Based DNA Methylation as Biomarker for Breast Cancer: A Systematic Review. Clin. Epigenetics.

[2] Chan S.C.H., Liang J.Q. (2022). Advances in Tests for Colorectal Cancer Screening and Diagnosis. Expert Rev. Mol. Diagn..

[3] Tobi E.W., Slieker R.C., Luijk R., Dekkers K.F., Stein A.D., Xu K.M., Slagboom P.E., van Zwet E.W., Lumey L.H., Biobank-based Integrative Omics Studies Consortium (2018). DNA Methylation as a Mediator of the Association between Prenatal Adversity and Risk Factors for Metabolic Disease in Adulthood. Sci. Adv..

[4] Shukla S., Ying W., Gray F., Yao Y., Simes M.L., Zhao Q., Miao H., Cho H.J., González-Alonso P., Winkler A. (2021). Small-Molecule Inhibitors Targeting Polycomb Repressive Complex 1 RING Domain. Nat. Chem. Biol..

[5] Vidal M., Starowicz K. (2017). Polycomb Complexes PRC1 and Their Function in Hematopoiesis. Exp. Hematol..

[6] Ma R., Zhang Y., Sun T., Cheng B. (2014). Epigenetic Regulation by Polycomb Group Complexes: Focus on Roles of CBX Proteins. J. Zhejiang Univ. Sci. B.

[7] Jaensch E.S., Zhu J., Cochrane J.C., Marr S.K., Oei T.A., Damle M., McCaslin E.Z., Kingston R.E. (2021). A Polycomb Domain Found in Committed Cells Impairs Differentiation When Introduced into PRC1 in Pluripotent Cells. Mol. Cell.

[8] Vincenz C., Kerppola T.K. (2008). Different Polycomb Group CBX Family Proteins Associate with Distinct Regions of Chromatin Using Nonhomologous Protein Sequences. Proc. Natl. Acad. Sci. USA.

[9] Zhang X., Zhou W., Zhang Y., Liu Z. (2022). CBX3 Is a Prognostic Biomarker Correlated with ATR Activation and Immune Infiltration in Head and Neck Squamous Cell Carcinoma. Int. J. Gen. Med..

[10] Xie X., Ning Y., Long J., Wang H., Chen X. (2020). Diverse CBX Family Members as Potential Prognostic Biomarkers in Non-Small-Cell Lung Cancer. FEBS Open Bio.

[11] Nielsen A.L., Ortiz J.A., You J., Oulad-Abdelghani M., Khechumian R., Gansmuller A., Chambon P., Losson R. (1999). Interaction with Members of the Heterochromatin Protein 1 (HP1) Family and Histone Deacetylation Are Differentially Involved in Transcriptional Silencing by Members of the TIF1 Family. EMBO J..

[12] Saunders W.S., Chue C., Goebl M., Craig C., Clark R.F., Powers J.A., Eissenberg J.C., Elgin S.C., Rothfield N.F., Earnshaw W.C. (1993). Molecular Cloning of a Human Homologue of Drosophila Heterochromatin Protein HP1 Using Anti-Centromere Autoantibodies with Anti-Chromo Specificity. J. Cell Sci..

[13] Paro R., Hogness D.S. (1991). The Polycomb Protein Shares a Homologous Domain with a Heterochromatin-Associated Protein of Drosophila. Proc. Natl. Acad. Sci. USA.

[14] Lachner M., O’Carroll D., Rea S., Mechtler K., Jenuwein T. (2001). Methylation of Histone H3 Lysine 9 Creates a Binding Site for HP1 Proteins. Nature.

[15] Nielsen A.L., Oulad-Abdelghani M., Ortiz J.A., Remboutsika E., Chambon P., Losson R. (2001). Heterochromatin Formation in Mammalian Cells: Interaction between Histones and HP1 Proteins. Mol. Cell.

[16] Aasland R., Stewart A.F. (1995). The Chromo Shadow Domain, a Second Chromo Domain in Heterochromatin-Binding Protein 1, HP1. Nucleic Acids Res..

[17] Cowieson N.P., Partridge J.F., Allshire R.C., McLaughlin P.J. (2000). Dimerisation of a Chromo Shadow Domain and Distinctions from the Chromodomain as Revealed by Structural Analysis. Curr. Biol..

[18] Brasher S.V., Smith B.O., Fogh R.H., Nietlispach D., Thiru A., Nielsen P.R., Broadhurst R.W., Ball L.J., Murzina N.V., Laue E.D. (2000). The Structure of Mouse HP1 Suggests a Unique Mode of Single Peptide Recognition by the Shadow Chromo Domain Dimer. EMBO J..

[19] Ruan J., Ouyang H., Amaya M.F., Ravichandran M., Loppnau P., Min J., Zang J. (2012). Structural Basis of the Chromodomain of Cbx3 Bound to Methylated Peptides from Histone H1 and G9a. PLoS ONE.

[20] Ligresti G., Caporarello N., Meridew J.A., Jones D.L., Tan Q., Choi K.M., Haak A.J., Aravamudhan A., Roden A.C., Prakash Y.S. (2019). CBX5/G9a/H3K9me-Mediated Gene Repression Is Essential to Fibroblast Activation during Lung Fibrosis. JCI Insight.

[21] Casale A.M., Cappucci U., Fanti L., Piacentini L. (2019). Heterochromatin Protein 1 (HP1) Is Intrinsically Required for Post-Transcriptional Regulation of Drosophila Germline Stem Cell (GSC) Maintenance. Sci. Rep..

[22] Azzaz A.M., Vitalini M.W., Thomas A.S., Price J.P., Blacketer M.J., Cryderman D.E., Zirbel L.N., Woodcock C.L., Elcock A.H., Wallrath L.L. (2014). Human Heterochromatin Protein 1α Promotes Nucleosome Associations That Drive Chromatin Condensation. J. Biol. Chem..

[23] Jacobs S.A., Khorasanizadeh S. (2002). Structure of HP1 Chromodomain Bound to a Lysine 9-Methylated Histone H3 Tail. Science.

[24] Lomberk G., Wallrath L., Urrutia R. (2006). The Heterochromatin Protein 1 Family. Genome Biol..

[25] Fan Y., Li H., Liang X., Xiang Z. (2017). CBX3 Promotes Colon Cancer Cell Proliferation by CDK6 Kinase-Independent Function during Cell Cycle. Oncotarget.

[26] Smallwood A., Hon G.C., Jin F., Henry R.E., Espinosa J.M., Ren B. (2012). CBX3 Regulates Efficient RNA Processing Genome-Wide. Genome Res..

[27] Niu H., Chen P., Fan L., Sun B. (2022). Comprehensive Pan-Cancer Analysis on CBX3 as a Prognostic and Immunological Biomarker. BMC Med. Genom..

[28] Huang C., Su T., Xue Y., Cheng C., Lay F.D., McKee R.A., Li M., Vashisht A., Wohlschlegel J., Novitch B.G. (2017). Cbx3 Maintains Lineage Specificity during Neural Differentiation. Genes Dev..

[29] Zhao Z., Shilatifard A. (2019). Epigenetic Modifications of Histones in Cancer. Genome Biol..

[30] Saldi T., Cortazar M.A., Sheridan R.M., Bentley D.L. (2016). Coupling of RNA Polymerase II Transcription Elongation with Pre-MRNA Splicing. J. Mol. Biol..

[31] Sheldon L.A. (2017). Inhibition of E2F1 Activity and Cell Cycle Progression by Arsenic via Retinoblastoma Protein. Cell Cycle.

[32] Pei Y., Banerjee S., Sun Z., Jha H.C., Saha A., Robertson E.S. (2016). EBV Nuclear Antigen 3C Mediates Regulation of E2F6 to Inhibit E2F1 Transcription and Promote Cell Proliferation. PLoS Pathog..

[33] Marei H.E., Althani A., Afifi N., Hasan A., Caceci T., Pozzoli G., Morrione A., Giordano A., Cenciarelli C. (2021). P53 Signaling in Cancer Progression and Therapy. Cancer Cell Int..

[34] Wang Z., Jia R., Wang L., Yang Q., Hu X., Fu Q., Zhang X., Li W., Ren Y. (2022). The Emerging Roles of Rad51 in Cancer and Its Potential as a Therapeutic Target. Front. Oncol..

[35] Orhan E., Velazquez C., Tabet I., Sardet C., Theillet C. (2021). Regulation of RAD51 at the Transcriptional and Functional Levels: What Prospects for Cancer Therapy?. Cancers.

[36] Li E., Xia M., Du Y., Long K., Ji F., Pan F., He L., Hu Z., Guo Z. (2022). METTL3 Promotes Homologous Recombination Repair and Modulates Chemotherapeutic Response in Breast Cancer by Regulating the EGF/RAD51 Axis. Elife.

[37] Wang H., Zhao W., Wang J., Zhang Z. (2022). Clinicopathological Significance of CBX3 in Colorectal Cancer: An Intensive Expression Study Based on Formalin-fixed and Paraffin-embedded Tissues. Pathol. Int..

[38] Yang H., Pu L., Li R., Zhu R. (2023). NCAPG Is Transcriptionally Regulated by CBX3 and Activates the Wnt/β-Catenin Signaling Pathway to Promote Proliferation and the Cell Cycle and Inhibit Apoptosis in Colorectal Cancer. J. Gastrointest. Oncol..

[39] Liu J., Zhan Y., Wang J., Wang J., Guo J., Kong D. (2020). LncRNA-SNHG17 Promotes Colon Adenocarcinoma Progression and Serves as a Sponge for MiR-375 to Regulate CBX3 Expression. Am. J. Transl. Res..

[40] Zhang P., Yang X., Zha Z., Zhu Y., Zhang G., Li G. (2022). CBX3 Regulated by MiR-139 Promotes the Development of HCC by Regulating Cell Cycle Progression. Cell Cycle.

[41] Beermann J., Piccoli M.-T., Viereck J., Thum T. (2016). Non-Coding RNAs in Development and Disease: Background, Mechanisms, and Therapeutic Approaches. Physiol. Rev..

[42] Farooqi A.A., Fayyaz S., Poltronieri P., Calin G., Mallardo M. (2022). Epigenetic Deregulation in Cancer: Enzyme Players and Non-Coding RNAs. Semin. Cancer Biol..

[43] Miguel V., Lamas S., Espinosa-Diez C. (2020). Role of Non-Coding-RNAs in Response to Environmental Stressors and Consequences on Human Health. Redox Biol..

[44] Skalska L., Begley V., Beltran M., Lukauskas S., Khandelwal G., Faull P., Bhamra A., Tavares M., Wellman R., Tvardovskiy A. (2021). Nascent RNA Antagonizes the Interaction of a Set of Regulatory Proteins with Chromatin. Mol. Cell.

[45] Burke M.J., Bhatla T. (2014). Epigenetic Modifications in Pediatric Acute Lymphoblastic Leukemia. Front. Pediatr..

[46] Handy D.E., Castro R., Loscalzo J. (2011). Epigenetic Modifications: Basic Mechanisms and Role in Cardiovascular Disease. Circulation.

[47] Lin H., Lian J., Xia L., Guan G., You J. (2020). CBX3 Promotes Gastric Cancer Progression and Affects Factors Related to Immunotherapeutic Responses. Cancer Manag. Res..

[48] Ma C., Nie X.G., Wang Y.L., Liu X.H., Liang X., Zhou Q.L., Wu D.P. (2019). CBX3 Predicts an Unfavorable Prognosis and Promotes Tumorigenesis in Osteosarcoma. Mol. Med. Rep..

[49] Alam H., Li N., Dhar S.S., Wu S.J., Lv J., Chen K., Flores E.R., Baseler L., Lee M.G. (2018). HP1γ Promotes Lung Adenocarcinoma by Downregulating the Transcription-Repressive Regulators NCOR2 and ZBTB7A. Cancer Res..

[50] Lin H., Zhao X., Xia L., Lian J., You J. (2020). Clinicopathological and Prognostic Significance of CBX3 Expression in Human Cancer: A Systematic Review and Meta-Analysis. Dis. Markers.

[51] Chang S.C., Lai Y.C., Chen Y.C., Wang N.K., Wang W.S., Lai J.I. (2018). CBX3/Heterochromatin Protein 1 Gamma Is Significantly Upregulated in Patients with Non-Small Cell Lung Cancer. Asia-Pac. J. Clin. Oncol..

[52] Jin X., Zhang B., Zhang H., Yu H. (2022). Smoking-Associated Upregulation of CBX3 Suppresses ARHGAP24 Expression to Activate Rac1 Signaling and Promote Tumor Progression in Lung Adenocarcinoma. Oncogene.

[53] Wang J., Yang B., Zhang X., Liu S., Pan X., Ma C., Ma S., Yu D., Wu W. (2023). Chromobox Proteins in Cancer: Multifaceted Functions and Strategies for Modulation (Review). Int. J. Oncol..

[54] Zhang C., Chang L., Yao Y., Chao C., Ge Z., Fan C., Yu H., Wang B., Yang J. (2021). Role of the CBX Molecular Family in Lung Adenocarcinoma Tumorigenesis and Immune Infiltration. Front. Genet..

[55] Hu K., Yao L., Xu Z., Yan Y., Li J. (2022). Prognostic Value and Therapeutic Potential of CBX Family Members in Ovarian Cancer. Front. Cell Dev. Biol..

[56] Czerwinska P., Mackiewicz A.A. (2022). Mining Transcriptomic Data to Uncover the Association between CBX Family Members and Cancer Stemness. Int. J. Mol. Sci..

[57] Sundar I.K., Nevid M.Z., Friedman A.E., Rahman I. (2014). Cigarette Smoke Induces Distinct Histone Modifications in Lung Cells: Implications for the Pathogenesis of COPD and Lung Cancer. J. Proteome Res..

[58] Zhang H., Yu H., Ren D., Sun Y., Guo F., Cai H., Zhou C., Zhou Y., Jin X., Wu H. (2022). CBX3 Regulated by YBX1 Promotes Smoking-Induced Pancreatic Cancer Progression via Inhibiting SMURF2 Expression. Int. J. Biol. Sci..

[59] Haley J.A., Haughney E., Ullman E., Bean J., Haley J.D., Fink M.Y. (2014). Altered Transcriptional Control Networks with Trans-Differentiation of Isogenic Mutant-KRas NSCLC Models. Front. Oncol..

[60] Bosso G., Cipressa F., Tullo L., Cenci G. (2023). Co-Amplification of CBX3 with EGFR or RAC1 in Human Cancers Corroborated by a Conserved Genetic Interaction among the Genes. Cell Death Discov..

[61] Xu H., Jiang C., Chen D., Wu Y., Lu J., Zhong L., Yao F. (2022). Analysis of Pan-Cancer Revealed the Immunological and Prognostic Potential of CBX3 in Human Tumors. Front. Med..

[62] Chen J., Lin Y., Zheng S., Chen Q., Tang S., Zhong X. (2023). CBX3 Promotes Clear Cell Renal Carcinoma through PI3K/AKT Activation and Aberrant Immunity. J. Transl. Med..

[63] Chen H., Han C., Liu D., Wang F., Ha C. (2022). CBX3 Promotes Ovarian Cancer Progression by Regulating P53/P21-Mediated Glucose Metabolism via Inhibiting NCOR2. Arch. Med. Sci..

[64] Taguchi Y.-H., Wang H. (2017). Genetic Association between Amyotrophic Lateral Sclerosis and Cancer. Genes.

[65] Loboda A., Nebozhyn M., Klinghoffer R., Frazier J., Chastain M., Arthur W., Roberts B., Zhang T., Chenard M., Haines B. (2010). A Gene Expression Signature of RAS Pathway Dependence Predicts Response to PI3K and RAS Pathway Inhibitors and Expands the Population of RAS Pathway Activated Tumors. BMC Med. Genom..

[66] Pavan A., Bragadin A.B., Calvetti L., Ferro A., Zulato E., Attili I., Nardo G., Dal Maso A., Frega S., Menin A.G. (2021). Role of next Generation Sequencing-Based Liquid Biopsy in Advanced Non-Small Cell Lung Cancer Patients Treated with Immune Checkpoint Inhibitors: Impact of STK11, KRAS and TP53 Mutations and Co-Mutations on Outcome. Transl. Lung Cancer Res..

[67] de Jesus V.H.F., Mathias-Machado M.C., de Farias J.P.F., Aruquipa M.P.S., Jácome A.A., Peixoto R.D. (2023). Targeting KRAS in Pancreatic Ductal Adenocarcinoma: The Long Road to Cure. Cancers.

[68] Kalungi F., Nsubuga A., Anywar G. (2023). Network Analysis and Molecular Docking Studies of Quercetin as a Potential Treatment for Prostate Cancer. In Silico Pharmacol..

[69] Mukhopadhyay S., Huang H.-Y., Lin Z., Ranieri M., Li S., Sahu S., Liu Y., Ban Y., Guidry K., Hu H. (2023). Genome-Wide CRISPR Screens Identify Multiple Synthetic Lethal Targets That Enhance KRASG12C Inhibitor Efficacy. Cancer Res..

[70] Hagenbeek T.J., Zbieg J.R., Hafner M., Mroue R., Lacap J.A., Sodir N.M., Noland C.L., Afghani S., Kishore A., Bhat K.P. (2023). An Allosteric Pan-TEAD Inhibitor Blocks Oncogenic YAP/TAZ Signaling and Overcomes KRAS G12C Inhibitor Resistance. Nat. Cancer.

[71] Cluntun A.A., Lukey M.J., Cerione R.A., Locasale J.W. (2017). Glutamine Metabolism in Cancer: Understanding the Heterogeneity. Trends Cancer.

[72] Zhong X., Ni J., Jia Z., Yan H., Zhang Y., Liu Y. (2022). CBX3 Is Associated with Metastasis and Glutathione/Glycosphingolipid Metabolism in Colon Adenocarcinoma. J. Gastrointest. Oncol..

[73] Mukhopadhyay S., Saqcena M., Foster D.A. (2015). Synthetic Lethality in KRas-Driven Cancer Cells Created by Glutamine Deprivation. Oncoscience.

[74] Kolch W., Berta D., Rosta E. (2023). Dynamic Regulation of RAS and RAS Signaling. Biochem. J..

[75] Lim J.S., Ibaseta A., Fischer M.M., Cancilla B., O’Young G., Cristea S., Luca V.C., Yang D., Jahchan N.S., Hamard C. (2017). Intratumoural Heterogeneity Generated by Notch Signalling Promotes Small-Cell Lung Cancer. Nature.

[76] Zhang C., Chen D., Maguire E.M., He S., Chen J., An W., Yang M., Afzal T.A., Luong L.A., Zhang L. (2018). Cbx3 Inhibits Vascular Smooth Muscle Cell Proliferation, Migration, and Neointima Formation. Cardiovasc. Res..

[77] Kopan R. (2012). Notch Signaling. Cold Spring Harb. Perspect. Biol..

[78] Zhou B., Lin W., Long Y., Yang Y., Zhang H., Wu K., Chu Q. (2022). Notch Signaling Pathway: Architecture, Disease, and Therapeutics. Signal Transduct. Target Ther..

[79] Ragot H., Monfort A., Baudet M., Azibani F., Fazal L., Merval R., Polidano E., Cohen-Solal A., Delcayre C., Vodovar N. (2016). Loss of Notch3 Signaling in Vascular Smooth Muscle Cells Promotes Severe Heart Failure Upon Hypertension. Hypertension.

[80] Zou B., Zhou X.-L., Lai S.-Q., Liu J.-C. (2018). Notch Signaling and Non-Small Cell Lung Cancer. Oncol. Lett..

[81] Janghorban M., Xin L., Rosen J.M., Zhang X.H.-F. (2018). Notch Signaling as a Regulator of the Tumor Immune Response: To Target or Not to Target?. Front. Immunol..

[82] Sorrentino C., Cuneo A., Roti G. (2019). Therapeutic Targeting of Notch Signaling Pathway in Hematological Malignancies. Mediterr. J. Hematol. Infect. Dis..

[83] Koni M., Pinnarò V., Brizzi M.F. (2020). The Wnt Signalling Pathway: A Tailored Target in Cancer. Int. J. Mol. Sci..

[84] Mauser R., Kungulovski G., Keup C., Reinhardt R., Jeltsch A. (2017). Application of Dual Reading Domains as Novel Reagents in Chromatin Biology Reveals a New H3K9me3 and H3K36me2/3 Bivalent Chromatin State. Epigenetics Chromatin.

[85] Chen L.-Y., Cheng C.-S., Qu C., Wang P., Chen H., Meng Z.-Q., Chen Z. (2018). Overexpression of CBX3 in Pancreatic Adenocarcinoma Promotes Cell Cycle Transition-Associated Tumor Progression. Int. J. Mol. Sci..

[86] Saxena K., Konopleva M. (2020). An Expert Overview of Emerging Therapies for Acute Myeloid Leukemia: Novel Small Molecules Targeting Apoptosis, P53, Transcriptional Regulation and Metabolism. Expert Opin. Investig. Drugs.

[87] Sandy Z., da Costa I.C., Schmidt C.K. (2020). More than Meets the ISG15: Emerging Roles in the DNA Damage Response and Beyond. Biomolecules.

[88] Carr S.M., Munro S., La Thangue N.B. (2012). Lysine Methylation and the Regulation of P53. Essays Biochem..

[89] Yang B., Yang N., Chen Y., Zhu M., Lian Y., Xiong Z., Wang B., Feng L., Jia X. (2020). An Integrated Strategy for Effective-Component Discovery of Astragali Radix in the Treatment of Lung Cancer. Front. Pharmacol..

[90] Hirata H., Hinoda Y., Kikuno N., Kawamoto K., Suehiro Y., Tanaka Y., Dahiya R. (2007). MDM2 SNP309 Polymorphism as Risk Factor for Susceptibility and Poor Prognosis in Renal Cell Carcinoma. Clin. Cancer Res..

[91] Appert-Collin A., Hubert P., Crémel G., Bennasroune A. (2015). Role of ErbB Receptors in Cancer Cell Migration and Invasion. Front. Pharmacol..

[92] Fry W.H.D., Kotelawala L., Sweeney C., Carraway K.L. (2009). Mechanisms of ErbB Receptor Negative Regulation and Relevance in Cancer. Exp. Cell Res..

[93] Black L.E., Longo J.F., Carroll S.L. (2019). Mechanisms of Receptor Tyrosine-Protein Kinase ErbB-3 (ERBB3) Action in Human Neoplasia. Am. J. Pathol..

[94] Cragg M.S., Kuroda J., Puthalakath H., Huang D.C.S., Strasser A. (2007). Gefitinib-Induced Killing of NSCLC Cell Lines Expressing Mutant EGFR Requires BIM and Can Be Enhanced by BH3 Mimetics. PLoS Med..

[95] Wang Z. (2017). ErbB Receptors and Cancer. Methods Mol. Biol..

[96] Uimari O., Rahmioglu N., Nyholt D.R., Vincent K., Missmer S.A., Becker C., Morris A.P., Montgomery G.W., Zondervan K.T. (2017). Genome-Wide Genetic Analyses Highlight Mitogen-Activated Protein Kinase (MAPK) Signaling in the Pathogenesis of Endometriosis. Hum. Reprod..

[97] Thandra K.C., Barsouk A., Saginala K., Aluru J.S., Barsouk A. (2021). Epidemiology of Lung Cancer. Contemp. Oncol..

[98] Lao Y., Shen D., Zhang W., He R., Jiang M. (2022). Immune Checkpoint Inhibitors in Cancer Therapy-How to Overcome Drug Resistance?. Cancers.

